# Impulsivity-based thrifty eating phenotype and the protective role of n-3 PUFAs intake in adolescents

**DOI:** 10.1038/tp.2016.16

**Published:** 2016-03-15

**Authors:** R S Reis, R Dalle Molle, T D Machado, A B Mucellini, D M Rodrigues, A Bortoluzzi, S M Bigonha, R Toazza, G A Salum, L Minuzzi, A Buchweitz, A R Franco, M C G Pelúzio, G G Manfro, P P Silveira

**Affiliations:** 1 Programa de Pós-Graduação em Saúde da Criança e do Adolescente, Departamento de Pediatria, Faculdade de Medicina, Hospital de Clínicas de Porto Alegre, Universidade Federal do Rio Grande do Sul, Porto Alegre, Brazil; 2 Programa de Pós-Graduação em Ciências Médicas: Psiquiatria, Faculdade de Medicina, Hospital de Clínicas de Porto Alegre, Universidade Federal do Rio Grande do Sul, Porto Alegre, Brazil; 3 Programa de Pós-Graduação em Neurociências, Instituto de Ciências Básicas da Saúde, Universidade Federal do Rio Grande do Sul, Porto Alegre, Brazil; 4 Programa de Pós-Graduação em Ciência da Nutrição, Departamento de Nutrição e Saúde, Universidade Federal de Viçosa, Viçosa, Brazil; 5 Department of Psychiatry and Behavioural Neurosciences, McMaster University, Hamilton, ON, Canada; 6 Instituto do Cérebro, Pontifícia Universidade Católica do Rio Grande do Sul, Porto Alegre, Brazil; 7 Programa de Pós-Graduação em Medicina e Ciências da Saúde, Faculdade de Medicina, Pontifícia Universidade Católica do Rio Grande do Sul, Porto Alegre, Brazil; 8 Programa de Pós-Graduação em Letras, Linguística, Faculdade de Letras, Pontifícia Universidade Católica do Rio Grande do Sul, Porto Alegre, Brazil; 9 Programa de Pós-Graduação em Engenharia Elétrica, Faculdade de Engenharia, Pontifícia Universidade Católica do Rio Grande do Sul, Porto Alegre, Brazil

## Abstract

The goal of the present study was to investigate whether intrauterine growth restriction (IUGR) affects brain responses to palatable foods and whether docosahexaenoic acid (DHA, an omega-3 fatty acid that is a primary structural component of the human brain) serum levels moderate the association between IUGR and brain and behavioral responses to palatable foods. Brain responses to palatable foods were investigated using a functional magnetic resonance imaging task in which participants were shown palatable foods, neutral foods and non-food items. Serum DHA was quantified in blood samples, and birth weight ratio (BWR) was used as a proxy for IUGR. The Dutch Eating Behavior Questionnaire (DEBQ) was used to evaluate eating behaviors. In the contrast palatable food > neutral items, we found an activation in the right superior frontal gyrus with BWR as the most important predictor; the lower the BWR (indicative of IUGR), the greater the activation of this region involved in impulse control/decision making facing the viewing of palatable food pictures versus neutral items. At the behavioral level, a general linear model predicting external eating using the DEBQ showed a significant interaction between DHA and IUGR status; in IUGR individuals, the higher the serum DHA, the lower is external eating. In conclusion, we suggest that IUGR moderates brain responses when facing stimuli related to palatable foods, activating an area related to impulse control. Moreover, higher intake of n-3 PUFAs can protect IUGR individuals from developing inappropriate eating behaviors, the putative mechanism of protection would involve decreasing intake in response to external food cues in adolescents/young adults.

## Introduction

Individuals exposed to intrauterine growth restriction (IUGR) are particularly vulnerable to the risk for chronic diseases in adulthood.^1^ In the long term, IUGR is associated with increased risk for obesity,^2, 3^ cardiovascular disease,^4^ type 2 diabetes,^1^ psychiatric disorders and altered neuropsychological functions.^5^

Our group and others have shown that IUGR is associated with changes in behavior and food preferences throughout life,^6^ spontaneously preferring palatable foods, rich in energy density but poor from a nutritional point of view.^7, 8, 9, 10, 11, 12^ These choices for specific types of food at different times during the life course can have an important role in increasing the risk for diseases widely described in these individuals.^13^ We have also shown that impulsivity and poor inhibitory control are important behavioral traits that moderate non-adaptive feeding in IUGR children.^14, 15^ Moreover, poor inhibitory control for food-related response is associated with higher body mass indices.^16^ Therefore, we hypothesized that molecules involved in the modulation of impulse control could have a protective role improving feeding behaviors specifically in IUGR children.

We recently showed that deficits in early inhibitory control predicted later food fussiness, and higher intakes of n-3 polyunsaturated fatty acids (n-3 PUFAs) in infancy protect IUGR children from developing food fussiness at 6 years of age.^15^ Arachidonic acid (AA; 20:4n-6) and docosahexaenoic acid (DHA; 22:6n-3) are essential for the brain growth and cognitive development; they accumulate rapidly in the brain during the later stages of gestation and early postnatal life.^17^ After the neonatal period, the major source of DHA is the diet,^18^ including vegetable oils, fish oils and red meat.^19, 20^ N-3 PUFAs deficiency increases reward sensitivity, impulsivity and sucrose solution intake in rodents.^21, 22^ Primarily, n-3 PUFAs show differential modulation of the mesocorticolimbic dopamine (DA) pathway; they are associated with reduced mesolimbic DA and increased mesocortical DA pathway activation.^23^ Infants with IUGR have impaired formation of DHA^24^ and thus are more likely to be vulnerable to the effects of subtle variations in nutritional supplies.

In the present study, we hypothesized that (1) IUGR affects brain responses to palatable foods in a brain functional magnetic resonance imaging (fMRI) task and (2) DHA serum concentrations could moderate the association between IUGR and brain and/or behavioral responses to palatable foods, decreasing non-adaptive behaviors.

## Materials and methods

### Participants

The study included 48 adolescents and young adults followed in a prospective cohort from the city of Porto Alegre, Brazil; the participants were followed in a prospective cohort. Details about this cohort can be found elsewhere.^25^ These participants were recruited from a community sample, selected from six schools belonging to a family health unit located in the city center. In 2008, children and adolescents from these schools were invited to participate in the study, which included psychiatric and nutritional assessments.^25^ A total of 242 individuals completed the assessment in 2008 and from this initial group, 75 participated (in 2013/2014) in a more in-depth re-evaluation that included psychiatric diagnosis, nutritional assessment, DNA extraction and an fMRI exam. The present study focused on fatty acid methyl esters, eating behaviors and fMRI measures. A total of 48 individuals had serum DHA and questionnaires available and from those, 27 also had brain fMRI data for the current study. The project was approved by the Research Ethics Committee from HCPA (Hospital de Clínicas de Porto Alegre; CAAE number 5278112500005327, protocol number in GPPG 12-0254) and the Research Ethics Committee of the Pontifícia Universidade Católica do Rio Grande do Sul, in accordance with the Brazilian and international regulations. All the participants signed an informed consent form. Confidentiality with respect to the identity of participants was ensured.

### Measurements

#### Intrauterine growth restriction

Fetal growth was based on the birth weight ratio (BWR), which is the ratio between the infant birth weight and the mean birth weight, sex- and gestational age-specific for the local population.^26^ BWR was used either as a continuous variable or categorized into IUGR (those in the lower tertile of the BWR distribution) or non-IUGR (two superior tertiles).

#### Anthropometric assessment

The anthropometric assessment was performed at the research center during the morning by trained researchers. All the participants were fasting when the measurements were taken. Weight and height were measured using accurate and calibrated equipments (digital platform balance Toledo, São Paulo, Brazil and vertical stadiometer Harpenden, Holtain Limited, Crymych, UK). The measures were performed in duplicate, and the average value was adopted. Body mass index (BMI) was calculated as weight (kg) divided by height (m^2^).

#### Food consumption

The Food Frequency Questionnaire^27^ was used to evaluate the food frequencies and quantity of consumption reported by the participants and these measures were converted into daily equivalents. Nutrient values for each food item (including DHA and EPA (eicosapentaenoic acid) consumption) were calculated. We used the American Food Composition Table (USDA—National Nutrient Database for Standard Reference Release 26)^28^ for the types of fatty acids investigated in this study, because it has greater nutritional information about these acids. The quantitative analysis of macro and micronutrients consumption was also calculated.

#### Biochemical analysis

The participants provided biological material (blood) for biochemical analysis. Blood samples were collected in the morning after fasting for 12 h and centrifuged at 4000 r.p.m. for 10 min. The obtained serum was divided into aliquots and stored at −80 °C for further processing.

The lipids were extracted as recommended by Folch *et al.*^29^ and saponified and esterified as recommended by Hartman and Lago.^30^ The identification of the fatty acid methyl esters was performed by gas chromatography using the CG-17A Flame Detector model (FID), Shimadzu (GC-17A, Kyoto, Japan). For the recording and analysis of chromatograms, the device was attached to a notebook, using the GC Solution program. The compounds were separated and identified on a Carbowax capillary column (30 m × 0.25 mm). For the chromatographic separation, a sample of 1 μl was injected with the aid of 10 μl syringe Hamilton (Reno, NV, USA) in Split System=5. Nitrogen gas was used as carrier with programmed linear velocity to 37.8 cm s^−1^. The temperatures of the injector and detector were controlled isothermally at 220 °C and 240 °C. The initial column temperature was 200 °C (maintained for 2 min), increasing at 4 °C per minute up to 240 °C, in 20 min total analysis. The flow of carrier gas in the column was 1.0 ml min^−1^. The identification of compounds was performed by the corresponding standard retention time (EPA and DHA). The analyses were performed in the Nutritional Biochemistry Laboratory at the Universidade Federal de Viçosa, Viçosa, Brazil.

#### fMRI acquisition parameters

All the participants who attended the evaluation of Clinical Research Center - HCPA were invited to attend the functional brain neuroimaging. Through telephone contact, the participants were checked for the exclusion criteria (presence of metals in the body, brackets, recent tattoo and pregnancy), and the exam was scheduled at the Institute of Brain Pontifícia Universidade Católica do Rio Grande do Sul. Participants were asked to keep fasting for at least 4 h. About 30 min before the fMRI, they received a standard snack (cereal bar+box of juice=174 kcal, 39 g carbohydrate (90% of total calories), 0.9 g protein (2% of total calories) and 1.6 g of lipids (8% of total calories)).

Image acquisition was performed using a 3.0 Tesla scanner (GE Healthcare Signa HDxT, Waukesha, WI, USA), with an eight channels head coil for signal reception. Structural images: T1 with voxels in isotropic spatial resolution of 1mm^3^, 170 contiguous slices and matrix image of 256 × 256 (frequency and phase). Images were inversion recovery type with TE=2.18 ms and TR=−6.1 ms. Structural acquisition was followed by the acquisition of functional images.

The acquisition of fMRI was performed by acquiring echo-planar T2* images (EPI) BOLD with 26 axial slices interspersed with a slice thickness of 4.0 mm and gap of 0.4 mm, FOV of 240 mm × 240 mm and matrix size of 80 × 64, TE=30 ms, TR=2.000 ms, flip angle of 90°. The task was divided into three runs with 233 volumes; each run lasted 7 min and 46 s. During the fMRI scan, the participants performed a food palatability evaluation task.

#### fMRI design and stimuli

The fMRI study design was adapted from Page *et al.*^31^ The goal was to investigate the brain responses of participants who judged the palatability of highly palatable foods, neutral foods and neutral items (non-food items, such as chair, pencil, towel). Figures were selected from a database of images from Deluchi.^32^ and the International Affective Picture System.^33^ A pilot study that investigated adolescents from the same age range of the present study population was performed to norm which food items were perceived as highly palatable and neutral.

The paradigm was created and presented using the E-Prime software (version 2, Psychological Software Tools, Pittsburgh, PA, USA); it was divided into three blocks of approximately 7 min each. Each block included 21 randomized images (seven highly palatable foods, seven neutral foods and seven neutral items), resulting in a total of 63 pictures. Each food trial consisted of image presentation (4 s) followed by two probe questions: ‘How much do you like the food?' (5 s) and ‘How much do you want to eat the food now?' (5 s). Participants had responded, by pressing buttons, to a scale that ranged from 1 (zero) to 4 (very much) using an fMRI compatible button box. Inter-trial interval ranged from 3 to 9 s. Trials with neutral items followed the same procedures; the probe questions, however, asked: ‘What is the importance of the object to you?' and ‘How useful is the object to you?'. A practice session was carried out before the fMRI scan, in which participants were shown five different trials and given the task instructions. The instructions were repeated to participants in the scanner before each fMRI run.

#### fMRI data analyses

The images were pre-processed using the Statistical Parametric Mapping software (SPM8, University College London, UK; http://www.fil.ion.ucl.ac.uk/spm). Preprocessing included slice-time and motion.^34, 35^ Four participants were excluded due to excessive head movement during fMRI. The motion-corrected fMRI images were co-registred with the individual anatomical images (T1).^36^ The T1 images were segmented into the gray matter, white matter and the cerebrospinal fluid;^37^ and also spatially normalized to a standard space (MNI152 template). Using the same registration parameters for the T1 image, fMRI images were registered to the MNI152 space and then smoothed using an 8 × 8 × 8 mm Gaussian filter with full-width half maximum. Level 1 analysis was performed using the timing of the palatable, neutral, non-food items and baseline periods (convolved with an ideal homodynamic response curve) as regressors in a multiple regression analysis. Beta coefficients for each condition were then used for the group analysis.

### Brain responses to palatable foods associated with fetal growth

The second level takes into account the estimates of specific parameters of the subject and the first level of variance estimates.^38^ This is a group analysis.^39^ In this study, the second level was used to investigate brain activations, with the outcome brain activation and as predictors serum levels of DHA and fetal growth values (BWR). To compare the distribution of activation across the three experimental conditions (palatable, neutral and non-food items), two methods were used. First, whole-brain, voxel-wise analyses of variance was conducted to identify the areas responsive to the main effects of palatability, neutral and non-food items. Second, the *t*-test analyses were performed. For each condition, activation was assessed with *t*-tests using food type versus baseline contrast images (one per participant, per contrast). The activation for each condition, relative to baseline, is not reported. Next, for the contrasts between types of items, the brain activation for the palatable foods condition was contrasted with the brain activation for Neutral Food Items, and, subsequently, with non-food Items (we also contrasted neutral food versus non-food). Group analyses were performed using multiple regressions with a family-wise error correction for multiple comparisons (*P*<0.05) using BWR as an independent variable. All *t*-maps and *F*-maps were calculated for the entire cortical volume. To identify the anatomical classification of brain areas activated, the coordinates found by analyzing the second level (Statistical Parametric Mapping) were prepared and inserted manually in *x*, *y*, *z* in the Talairach client interface (http://www.talairach.org/).

### Interaction between IUGR and serum DHA on behavioral responses to palatable foods

Individuals responded to the Dutch Eating Behavior Questionnaire (DEBQ),^40^ which is an instrument that measures three dimensions of eating style (emotional eating, external eating and restrained eating). This 33-item instrument is rated on a five-point scale (from ‘never' to ‘very often'). The final score on each scale is the average of the item scores on that scale. The scales have good internal reliability and good construct and predictive validity.^41, 42, 43^ This instrument can be easily completed by adolescents,^44, 45^ it was validated for Portuguese,^46^ translated for the Brazilian population^47^ and recently used in a sample of university students from São Paulo.^48^

### Statistical methods

Data were entered and analyzed using the Statistical Package for Social Sciences (SPSS), version 22.0 (SPSS, IBM, Chicago, IL, USA). Descriptive statistics were performed comparing characteristics of individuals who participated and did not participate in 2013/2014 (including sex, race, maternal education and socioeconomic status, birth weight and BMI). These analyses were also performed to compare the individuals according to the presence or absence of IUGR at birth (also including: anxiety, age, BMI *z*-score, serum DHA and EPA concentration, and eating behavior domains of DEBQ), using chi-square tests (for categorical variables) and Student's *t*-tests (for continuous variables).

The primary analysis was based on the model proposed by Holmbeck^49^ consisting of a general linear model. The variables included IUGR (categorical) and serum DHA concentration (continuous); the outcome was the eating behavior measured by the DEBQ. The DEBQ analyses were adjusted for between-group differences in current BMI *z*-score, as there was a significant difference between groups (IUGR, non-IUGR) in the preliminary analysis of data. In all the analyses, we considered the significance level of 5%.

## Results

### Attrition analysis within the cohort

The participants in the current study were representative of the whole cohort evaluated in 2008, as no differences were seen in the main confounders. Individuals who participated or not in the current study were similar with regard to gender, skin color, maternal education, socioeconomic status, birth weight and current BMI (all *P*-values >0.05). When comparing the participants according to birth status, the mean±s.e.m. of serum DHA concentration was 4.25±0.34 (μg g^−1^) for non-IUGR and 3.74±0.32 (μg g^−1^) for IUGR individuals (Student's *t*-test=0.37). Except for expected differences in birth weight and BMI *z*-scores, there were no statistical differences in several other confounding variables between IUGR and non-IUGR individuals (see Table 1).

### Fetal growth modulation of brain response

**Figure 1:**
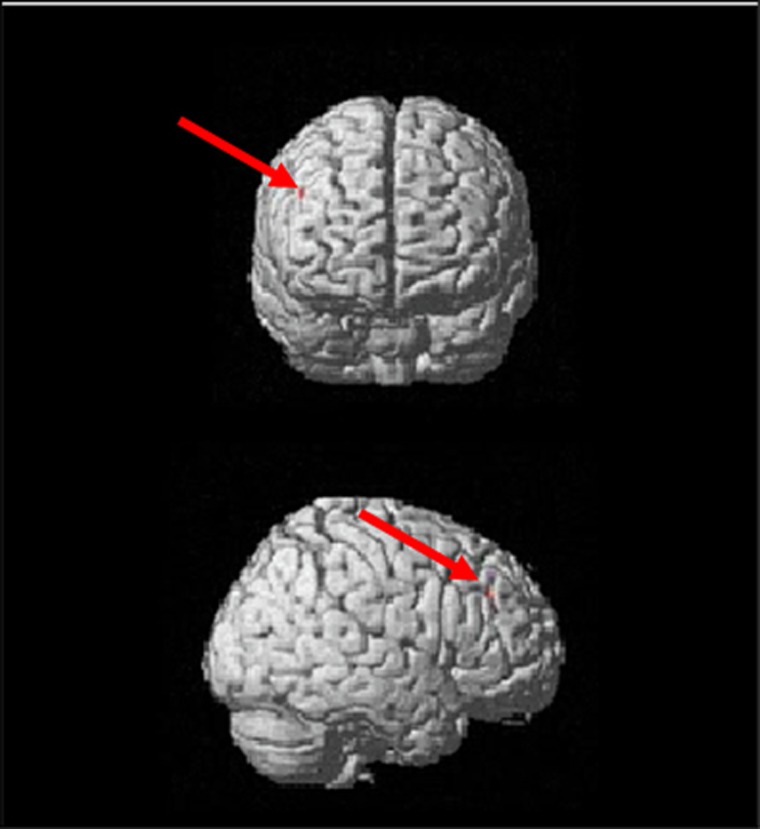
Activation of the right superior frontal gyrus for participants when having higher DHA serum levels and lower BWR values; the cluster was identified in the comparison of activation for palatable foods > neutral items (multiple regression (*P*<0.05 FWE correction for multiple comparisons; *T*=5.67); two voxels in cluster). BWR, birth weight ratio; DHA, docosahexaenoic acid; FWE, family-wise error.

Results show more activation of the right superior frontal gyrus (RSFG; *x*=46, *y*=38, *z*=32, voxels=2; and family-wise error corrected *P*=0.034) using serum DHA as positive and BWR as negative predictors (Figure 1). BWR is the most important predictor of brain activation for the RSFG activation, as shown by changing the direction of the predictors (Table 2). In other words, the lower the BWR (indicative of IUGR), the greater the RSFG activation. No significant activation was found for the contrasts palatable food > neutral food or neutral food > non-food items. Table 2 depicts the results of brain fMRI in the contrast palatable food > neutral items.

### Interaction between IUGR and serum DHA on behavioral responses to palatable foods

**Figure 2:**
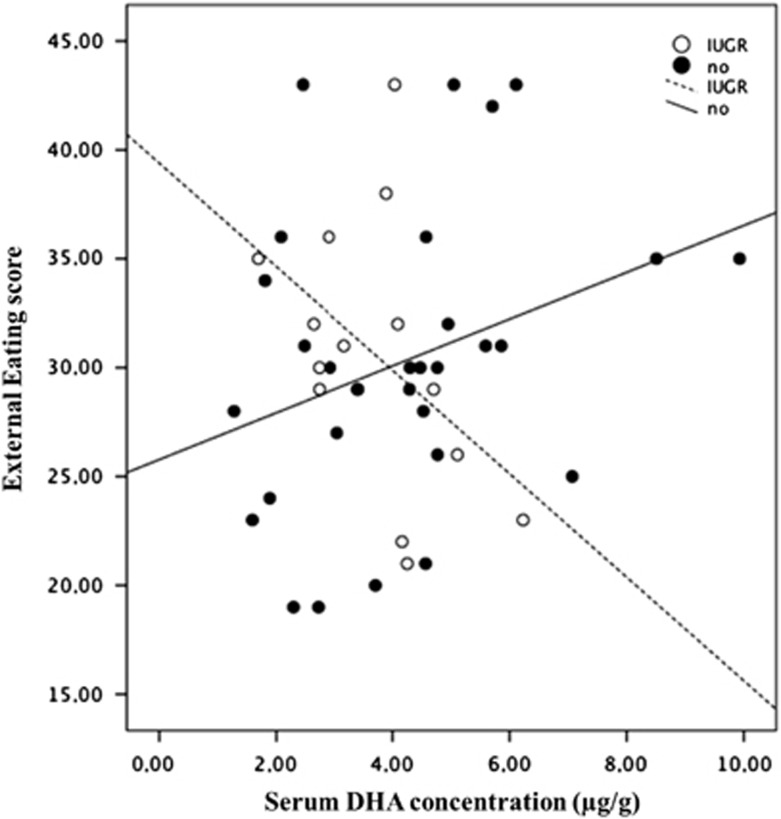
Interaction between IUGR and the serum DHA concentration on external eating domain in adolescents and young adults. DHA, docosahexaenoic acid; IUGR, intrauterine growth restriction.

A series of general linear models was performed for the different DEBQ scores measured. The general linear model predicting external eating showed a significant interaction between serum DHA concentration and IUGR status (yes/no) (Wald=5.845; df=1; and *P*=0.016) with main effects of IUGR (Wald=6.146; df=1; and *P*=0.013) and DHA (Wald=1.404; df=1; and *P*>0.05), Table 3 and Figure 2; that is, for that domain, in IUGR individuals, the more the DHA, the lower the score on external eating (*B*=−3.597; confidence interval=−6.514 to −0.681; *P*=0.016).

When considering sex as another variable in the general linear models, the results remained the same as described above (data not shown). There was no effect or interaction in the domain of DEBQ emotional eating (Wald=1.877; df=1; and *P*=0.171) with main effects of IUGR (Wald=2.934; df=1; and *P*=0.087) and DHA (Wald=0.520; df=1; and *P*=0.471), while the domain restrained eating did not reach statistical significance for the interaction (Wald=3.360; df=1; and *P*=0.067) with main effects of IUGR (Wald=6.082; df=1; and *P*=0.014) and DHA (Wald=5.196; df=1; and *P*=0.023; Table 3).

## Discussion

The results show that the degree of IUGR is associated with an increase in the activation of the right superior frontal gyrus, a brain area previously involved in processes of impulse control and decision making.^50, 51, 52, 53, 54^ Results also demonstrate that the serum level of DHA (a marker of n-3 PUFAs consumption) is inversely correlated with external eating scores in IUGR subjects, but not in normal birth weight subjects. This result suggests that at the behavioral level DHA has a role in decreasing impulsivity and therefore diminishing external eating in vulnerable individuals.

The relationship between impulsivity and food intake is mediated by external eating.^55^ It was recently shown that impulsive children eat more high-energy dense foods than lesser impulsive children, both before and after their lunch.^56^ Impulsivity is an important behavioral trait that links IUGR to palatable food intake and future higher BMI;^14^ decreasing external eating seems an important way by which n-3 PUFAs could protect IUGR individuals from increased palatable food intake and consequently developing obesity and its metabolic consequences. The external eating domain refers to the consumption in response to external stimuli related to food (vision, attractive smell of food), regardless of internal hunger and satiety signals,^57^ leading to food cravings^58, 59^ and obesity.^40, 57, 58, 59, 60^

In healthy subjects, the neural processing of visual stimuli food involves the primary and secondary sensory areas, limbic and paralimbic structures that respond to salient and reward processing, and prefrontal structures involved in cognitive control.^61, 62, 63^ Making judgments and decisions about food involves emotional and rational circuits.^63, 64, 65^ The neural response to food appears to be enhanced as a function of stimuli-salient properties (such as calorie content or hedonic properties)^66^ and of the degree of hunger.^63, 67^ The visual detection of food is the active anticipatory response that is likely to determine feeding behavior.^68^ In this sense, susceptibility to eating disorders may be related to the inability to process visual food stimuli appropriately.^63^ The present study shows that the degree of IUGR is associated with specific brain activation towards palatable food cues later in life.

In our study, DHA was not associated with significant modulation of brain activation, although it was associated with a behavioral effect (diminished external eating). It is possible that the sample size limited the ability to identify a central effect in the brain imaging data. It is also possible that the habitual consumption of n-3 PUFAs by the participants, reflected in the serum levels of DHA, may not be enough to meet the expected effect on brain, although our regional consumption of n-3 PUFAs is higher than in other regions of the country.^69, 70^ Supplementation with DHA for 8 weeks leads to an increase in functional activation of dorsolateral prefrontal regions during a sustained visual attention task in healthy children aged between 8 and 10 years, which corroborates the idea that the intake of DHA is a strong modulator of cortical function.^71^ Further studies exploring the effects of n-3 PUFAs supplementation on brain responses to palatable foods in IUGR individuals are warranted.

IUGR modulated the brain activity associated with judgment of palatable foods; activation for palatable foods relative to neutral foods was found in an area possibly associated with impulse control (superior frontal gyrus). Moreover, higher intake of n-3 PUFAs can protect individuals born with IUGR from developing inappropriate behaviors, especially decreasing intake in response to external food cues and adolescents/young adults. Future controlled, intervention studies are needed to inform about the clinical applications of this information, especially considering this specific population of individuals born with IUGR.

## Figures and Tables

**Table 1:** Study participants' baseline characteristics according to presence or absence of IUGR at birth

*Sample characteristics*	*Non-IUGR (*n=*33)*	*IUGR (*n=*14)*	P
Male (%)	12 (36.4%)	4 (28.6%)	0.74^a^
White skin color (%)	20 (60.6%)	7 (53.8%)	0.75^a^
Maternal education (⩽8 years) (%)	10 (50%)	2 (25%)	0.40^a^
Anxious (%)	13 (39.4%)	9 (64.3%)	0.20^a^
Age (years)	17.89±0.42	17.18±0.66	0.37^b^
ABEP score	2.5±0.16	2.5±0.23	1.0^b^
Birth weight (g)	3374.85±95.56	2636.43±107.92	<**0.0001**^b^
Body mass index (kg/m^2^)	23.51±0.72	21.06±1.15	0.073^b^
BMI *z*-score	0.59±0.19	−0.14±0.30	**0.046**^b^
Serum DHA concentration (μg g^−1^)	4.25±0.34	3.74±0.32	0.37^b^
Serum EPA concentration (μg g^−1^)	4.75±0.32	3.82±0.37	0.11^b^
DHA consumption (g)	0.14±0.04	0.10±0.05	0.59^b^
EPA consumption (g)	0.06±0.02	0.06±0.03	0.93^b^

Abbreviations: ABEP, *Associação Brasileira de Empresas de Pesquisa*; DHA, docosahexaenoic acid; EPA, eicosapentaenoic acid; IUGR, intrauterine growth restriction.

a Chi-square test.

b Student's *t*-test. Data are expressed as mean±s.e.m. or proportions (percentages).

One participant had no information about birth weight or gestational age and was therefore excluded. Significant results are presented in bold.

**Table 2:** Brain activation found in palatable food > neutral items, using multiple regression (FWE corrected for multiple comparisions, *P*<0.05)

*Contrast*	*Predictors*	*Outcome*	*Activation*
Palatable food > neutral items	↑ **Serum DHA concentration** ↓ **BWR**	Brain activation	**Right superior frontal gyrus** (*P*-corrected=0.034)
	↑ Serum DHA concentration ↓ serum DHA concentration		No activation found
	↑ BWR (birth weight ratio)		No activation found
	↓ **BWR** (birth weight ratio)		**Right superior frontal gyrus** (*P*-corrected=0.028)
	↓ **Serum DHA concentration** ↓ **BWR** (birth weight ratio)		**Right superior frontal gyrus** (*P*-corrected=0.025)

Abbreviations: BWR, birth weight ratio; DHA, docosahexaenoic acid; FWE, family-wise error. Significant results are presented in bold.

**Table 3:** DEBQ domains according to presence or absence of IUGR at birth

*DEBQ*	*Non-IUGR*	*IUGR*	P	*GLM*		
				*IUGR,* B *(CI)* P	*DHA,* B *(CI)* P	*Interaction,* P
Emotional eating	32.12±2.44	34.57±3.13	0.570^a^	21.551 (−3.109; 46.212) 0.087	0.972 (−1.251; 3.195) 0.471	0.171
External eating	30.12±1.15	30.50±1.68	0.856^a^	14.996 (3.140; 26.853) **0.013**	0.892 (−0.177; 1.961) 0.236	**0.016**
Restrained eating	22.09±1.96	26.50±3.39	0.243^a^	24.936 (5.118; 44.754) **0.014**	−0.637 (−2.423; 1.150) **0.023**	0.067

Abbreviations: CI, confidence interval; DEBQ, Dutch Eating Behavior Questionnaire; DHA, docosahexaenoic acid; GLM, general linear model; IUGR, intrauterine growth restriction.

a Student's *t*-test. Data are expressed as mean±s.e.m. Significant results are presented in bold.
